# Effect of Dietary Supplementation With Mixed Organic Acids on Immune Function, Antioxidative Characteristics, Digestive Enzymes Activity, and Intestinal Health in Broiler Chickens

**DOI:** 10.3389/fnut.2021.673316

**Published:** 2021-08-05

**Authors:** Jiayu Ma, Shad Mahfuz, Jian Wang, Xiangshu Piao

**Affiliations:** State Key Laboratory of Animal Nutrition, College of Animal Science and Technology, China Agricultural University, Beijing, China

**Keywords:** mixed organic acid, antioxidative characteristics, tight junction, intestinal health, broiler

## Abstract

The purpose of this study was to explore the effect of dietary supplementation with mixed organic acids on intestinal health, enzyme activity, and antioxidative characteristics in broilers. A total of 192 1-day-old chicks were evenly allocated to three experimental groups with eight replicates, a basal diet with 0 (Control), 3,000 mg/kg (LMOA), 6,000 mg/kg (HMOA) mixed organic acid. The tissue and serum samples were gathered on 21 and 42 d of the experiment. An increased (*P* < 0.05) concentration of IgA, D-lactate (D-LA), and interleukin-10 (IL-10) in the serum of broilers diets with HMOA was observed. The levels of total antioxidant capacity (T-AOC) and catalase activity (CAT) in serum were enhanced (*P* < 0.05) with dietary and mixed organic acid, respectively, and increased (*P* < 0.05) content of superoxide dismutase (SOD) and CAT in the duodenum of broilers diets with LMOA was noticed. Also, LMOA decreased (*P* < 0.05) the pH value of the duodenum and enhanced (*P* < 0.05) the amylase activity of the pancreas, the tight junction protein (mainly Claudin-1, Claudin-2, and ZO-1) in the duodenum of broilers fed with mixed organic acid were promoted (*P* < 0.05), and the LMOA group performed better in the small intestine. In cecum microbiota, LMOA and HMOA modulated the structure of microbiota and mainly reduced the relative abundance of *Escherichia coli*. In brief, dietary supplemented mixed organic acid improved the health status of broilers by promoting the immune function, enhancing the antioxidative characteristics and tight junction proteins expression as well as cecum microbiota. However, LMOA groups may be a better fit considering the comprehensive effects of experiments and economic costs.

## Introduction

With the accelerating industrialization of livestock farming and the increasing attention paid to food safety, companies are increasingly concerned about the problems associated with applying antibiotics in feed, such as drug resistance in pathogenic bacteria and drug residues in livestock products (1). Thus, reducing the application of antibiotics in feeds and finding alternatives is imperative to control the occurrence of diseases in livestock and reduce the harmful effects of antibiotics on humans. Consequently, the development and application of new green additives have emerged as a hot issue in feed research and industry (2). Mixed organic acids (MOA) are favored by feed and livestock enterprises because of their advantages of improving the growth performance and intestinal health of livestock, as well as the benefits of the three-free (i.e., drug resistance-free, residue-free, and pollution-free), which make the MOA the most promising green alternatives to antibiotics (3, 4). Numerous studies concentrated on the growth performance, diarrhea rate, the digestibility of nutrients, and intestinal health in swine (5–8) and poultry (9–11). However, limited literature has focused on the effects of high levels of MOA in diets on immune function, antioxidant properties, digestive enzymes activity as well as intestinal health of the broiler chickens. The objective of this experiment was to evaluate the effects of high and low levels of mixed organic acids in corn-soybean meal diets on immune parameters, antioxidant properties of serum and intestine, gastrointestinal enzyme activities, and intestinal health of broiler chickens to systematically and comprehensively assess the feeding effectiveness of mixed organic acids and understand the specific mechanism of organic acids as well as to provide a theoretical basis for researchers to analyze the feasibility of replacing antibiotics with mixed organic acid in livestock farming.

## Materials and Methods

This study was carried out in an experimental poultry shed at China Agricultural University. The proceedings of this research were licensed by the Institutional Animal Care and Use Committee of China Agricultural University (No.AW09089104-1, Beijing, China).

### Mixed Organic Acid Products

The mixed organic acid product named Fysal MP (product code: 2017SZ34) employed in the present study was sourced from Nutreco (Amsterdam, Netherlands), and it primarily contains formic acid (≥11%), formate ammonia (≥13%), propionate (≥10%), acetate (≥5.1%), lactate (≥4.2%), and other organic acids with lower concentrations ( ≤ 2%), such as malic acid and citric acid. The carrier is silica.

### Experimental House and Management

The poultry shed was designed with forced ventilation, radiant heating, and illumination. The chicks were housed in an average of 24 wire-mesh cages, each equipped with three automatic teat waterers and a round feeder (diameter: 37 cm), which provided 24 h of continuous light for the first 2 days before the chicks reached the poultry house. Thereafter, a 23-light:1-dark lighting regimen was implemented daily. During the 1st week of the experiment, the temperature was controlled at 34–35°C and gradually lowered by 1–2°C per week until the temperature was 25 ± 1°C while the relative humidity was held at 45–55%. All chicks were vaccinated with Newcastle disease and infectious bronchitis vaccine (LASOTA+H_120_, supplied by Qilu Animal Health Products Co., Ltd. Shandong, China) at d 7, d 14, and d 21 (the first two times by injection and the third time by mixing with drinking water), respectively.

### Chicks, Diets, and Experimental Procedures

A total of 192 1-day-old male chicks weighing 48.40 ± 0.64 g were obtained from commercial hatcheries (Arbor Acres Poultry Breeding Company, Beijing, China) and transferred to the experimental house in a van within 30 min at 35°C. After, 1-day-old male chicks were weighed on arrival at the experimental house and labeled with wing-rings. The chicks were randomly distributed into three experimental groups with eight replicates (cages) to receive a basal diet of corn-soybean meal supplemented with 0 (Ctrl), 3,000 mg/kg (low mixed organic acids, LMOA), and 6,000 mg/kg (high mixed organic acids, HMOA) of MOA, respectively. In total, eight replicates (cages) with eight chicks per treatment were designated for the present study. Table 1 lists the composition and nutritional levels of basal diets, which satisfied or excelled the NRC requirements. Additionally, the health status and the number of dead animals were observed and noted daily.

**Table 1:** Composition and nutrient levels of basal diets (DM^a^ basis).

**Ingredients (%)**	**Phase 1 (d 1 to d 21)**	**Phase 2 (d 22 to d 42)**
Corn, 8.2 % CP^a^	61.74	65.17
Soybean meal, 46% CP	28.50	24.50
Fish meal, 64.7% CP	3.29	3.33
Soy oil	2.90	3.81
Dicalcium phosphate	1.30	1.20
Limestone	1.28	1.10
Salt	0.30	0.30
L-lysine HCl, 78%	0.00	0.00
DL-Methionine, 98%	0.15	0.05
L-Threonine, 98%	0.04	0.04
Vitamin-mineral premix^b^	0.50	0.50
Total	100	100
**Nutritional levels^c^**		
Metabolizable energy, kcal/kg	3,050.73	3,150.11
Crude protein	20.68	19.06
Calcium	1.02	0.91
Digestible phosphorus	0.43	0.40
Standardized ileal digestible lysine	0.80	0.72
Standardized ileal digestible methionine	0.27	0.25
Standardized ileal digestible threonine	0.58	0.53

a *DM, dry matter; CP, crude protein*.

b *Premix provided the following per kg of feed: vitamin A, 10,000 IU; vitamin D_3_, 3,000 IU; vit-amin E, 24 mg; vitamin K_3_, 2.1 mg; vitamin B_12_, 2 mg; riboflavin, 5.0 mg; pantothenic acid, 15 mg; niacin, 40 mg; choline chloride, 500 mg; folic acid, 0.9 mg; vitamin B_6_, 3.0 mg; biotin, 0.05 mg; Mn (from MnSO_4_·H_2_O), 70 mg; Fe (from FeSO_4_·H_2_O), 80 mg; Zn (from ZnSO_4_·H_2_O), 100 mg; Cu (from CuSO_4_·5 H_2_O), 18.8 mg; I (from KI), 0.35 mg; Se (from Na_2_SeO_3_), 0.30 mg*.

c *The metabolizable energy and crude protein in nutrient levels were analyzed values, other nutrients were cacluated values*.

### Sampling Procedure

On the 21st and 42nd day, one broiler chicken with uniform weight was picked from each cage, respectively; 10 mL of the non-anticoagulant blood sample was drawn from the jugular vein into a vacutainer and placed at room temperature for 30 min, centrifuged 3,000 rpm at 4°C for 15 min, and the serum was prepared and stored at −20°C for the further determination of biochemical immune parameters.

Immediately after the chickens were slaughtered by cervical dislocation at the slaughterhouse, the gizzard, about 1/2 of the posterior segment of duodenum, jejunum, and the ileum were collected. The contents of the intestines were diluted 1:8 with de-ionized water, vibrated, and mixed with a small oscillator (250 r/min) for 5 min, then measured the pH value by a portable PHS-3C acidimeter (Precision Scientific Instruments Co., Ltd., Shanghai, China). Simultaneously, the liver, spleen, and pancreas samples were gathered to calculate the visceral weight index (Formulation: viscera weight index (%) = viscera weight/final body weight ^*^100%). Approximately 2 cm of jejunum, ileum, and duodenum at the 1/2 of the posterior segment were taken and cleaned the intestinal chyme gently with 0.9% normal saline, and the intestinal sample was collected and stored at −80 °C for determination of gene expression of tight junction protein and intestinal enzyme activity. Subsequently, we gathered the cecum chyme for the sequencing of the 16S rRNA gene.

### RNA Extraction and Real-Time Quantitative PCR

The Trizol reagent (Tiangen Biotech, Beijing, China) was applied to extract the total RNA of the small intestine according to the manufacturer's specifications, the content and purity of RNA were determined using a NanoDrop 2000 spectrophotometer (Thermo Fisher Scientific, Waltham, USA), and the integrity was assessed by agarose gel electrophoresis using ethidium bromide staining. The RNA to be measured was reverse-transcribed into cDNA by TransScript All-in-One First-Strand cDNA Synthesis SuperMIX for qPCR kit (QIAGEN, Northwestern Hilden, Germany). The reverse transcription (RT) reaction was composed of 0.5 μg total RNA, 5 μL 5× TransScript All-in-one SuperMix for qPCR, 0.5 μL gDNA Remover and 4 μL nuclease-free water (total in 10 μL). The reactions were conducted with the GeneAmp^®^ PCR System 9700 (Applied Biosystems, Waltham, USA) for 15 min at 42°C and 5 s at 85°C. The 10 μL RT reaction mixture was subsequently diluted ×10 with nuclease-free water and stored at −20°C. The LightCycler^®^ 480 II Real-time PCR Instrument (Roche, Basel, Swiss) was used and the PCR reaction mixture containing 3.6 μL nuclease-free water, 5 μL 2×PerfectStart^TM^ Green qPCR Master Mix, 0.5 μL forward primer (10 μmol/L), 0.5 μL reverse primer (10 μmol/L), and 1.0 μL cDNA. Reaction conditions (PCR efficiency: 94–105%) were 95°C and pre-denaturation for 1 min, followed by 45 cycles (denatured at 95°C for 15 s, annealed at 60°C for 30 s, and then fluorescence collected). Also, the product specificity was detected using a melting curve at the end of the cycle: a slow ramp-up from 60°C to 97°C with five fluorescence signal acquisitions per degree Celsius. The primers were designed and synthesized by Sangon Biotech (Shanghai, China) based on the mRNA sequences on the NCBI database (Table 2), the glyceraldehyde-3-phosphate dehydrogenase (GAPDH) gene was used as a housekeeping gene, the results of the relative quantitative expression were calculated using the comparative Ct methodology, the expression of the target gene was 2^−ΔΔCt^ [ΔΔCt = (Ct _target genes_-Ct _housekeeping genes_) experimental group-(Ct _target genes_-Ct _housekeeping genes_) control group], the analysis of all samples was repeated twice on the same PCR plate, and the data were averaged.

**Table 2:** Sequence and parameters of primers for PCR.

**Gene names**	**Primer sequence (5^**′**^-3^**′**^)**	**Length (bp)**
GAPDH	F: GAAGGCTGGGGCTCATCTG R: CAGTTGGTGGTGCACGATG	150
Occludin	F: AGATGGACAGCATCAACG R: CTGCCACATCCTGGTATT	90
Claudin-1	F: CCTCCACAGGGAAGGATTAC R: ACCTAGAGTCTGAACTCTGC	121
Claudin-2	F: CTCAGCCCTCCATCAAACA R: TGCTGCTGCTACACGTAT	82
ZO-1	F: TGGTACTGACCAACGTAGTTC R: AGGAGTGACATCTAATAAAGCG	94
ZO-2	F: ATGATAGCTGGTATGGTAGTCT R: CATGCGATCATCTGCGTC	113

*F, forward primer; R, reverse primer*.

### Serum Biochemical Immunity and Intestinal Enzyme Activity

Immunoglobulins (IgG, IgA), D-lactic acid (DLA), diamine oxidase (DAO), tumor necrosis factor-α (TNF-α), interleukin-1β (IL-1β), interleukin-6 (IL-6), and interleukin-10 (IL-10) in serum were detected via ELISA methodology, and the enzyme activity of amylase (Iodine-starch), lipase (P-nitrophenol ester), trypsin (benzoyl-DL-arginine-p-nitroanilide), and chymotrypsin (N-glutaryl-L-phenylalanine-p-nitroaniline) in the small intestine were measured by immunoturbidimetric methodology. Contents of total antioxidant capacity (T-AOC), malondialdehyde (MDA), catalase (CAT), glutathione peroxidase (GSH-Px), and superoxide dismutase (SOD) in serum, duodenum, jejunum, and ileum were examined via spectrophotometry (PU 8720 UV/VIS Scanning Spectrophotometer, Pye Unicam, UK). The commercial assay kits were supplied by Nanjing Jiancheng Institute of Bioengineering (Nanjing, China), and all the procedures were conducted according to the protocol of the kits.

### Pyrosequencing of 16S rRNA Amplicons

The cecum chyme was removed from the −80°C refrigerator and total DNA of the microbial community was extracted according to the instructions of DNeasy^®^ PowerSoil^®^ Pro Kit (QIAGEN, Northwestern Hilden, Germany), and the quality and purity of DNA were detected using a NanoDrop 2000 UV spectrophotometer (USA) and 1% agarose gel electrophoresis. The extracted DNA was diluted to 1 ng/μL with sterile water and used as a template for PCR amplification in the V3-V4 variable region of the 16S rRNA gene (12). Primer sequence: 338F (5'-ACTCCTACGGGAGGCAGCAG-3') and 806R (5'-GGACTACHVGGGTWTCTAAT-3'). Amplification procedure: 95°C pre-denaturation for 3 min, 27 cycles (95°C denaturation for 30 s, 55°C annealing for 30 s, 72°C extension for 30 s), and then stable extension at 72°C for 10 min. The final storage was performed at 4°C (PCR equipment: ABI GeneAmp^®^ 9700, Shanghai Aiyan Biological Technology, China). The PCR reaction system was as follows: 5× TransStart FastPfu buffer 4 μL, 2.5 mM dNTPs 2 μL, upstream primer (5 μM) 0.8 μL, downstream primer (5 μM) 0.8 μL, TransStart FastPfu DNA polymerase 0.4 μL, template DNA 10 ng, and added double-distilled water to 20 μL. The PCR products from the same sample were mixed and recovered using a 2% agarose gel, purified using the AxyPrep DNA Gel Extraction Kit (Axygen Biosciences, Union City, CA, USA), detected by 2% agarose gel electrophoresis, and quantified by Quantus™ Fluorometer (Promega, USA). The library construction was conducted by NEXTFLEX Rapid DNA-Seq Kit: (1) splice linkage; (2) removal of splice self-linked fragments using magnetic bead screening; (3) library template enrichment using PCR amplification; (4) recovery of PCR products by magnetic beads to obtain the final library. The quantification library was examined using a Qubit 2.0 fluorometer (Thermo Fisher Scientific, USA) to obtain a homogeneous concentration. Then sequenced on the Illumina's Miseq PE300 platform (Illumina, San Diego, USA) following the standard protocol to obtain 250 bp paired-end reads, which were assembled and filtered by PANDAseq to remove the tags with more than three ambiguous bases as well as the average quality score <20. The Raw data were deposited and uploaded to the NCBI SRA database (sequence number: PRJNA719231).

### Statistical Analysis

The raw data was preliminarily organized by Excel software (Microsoft, Redmond, USA). Data were analyzed by one-way ANOVA analysis using the GLM program of SAS 9.2 (SAS Institute Inc., Cary, NC, USA) in a completely randomized design. Each cage was used as an experimental unit for broiler growth performance, and the chosen broilers were considered as the experimental units for other properties. Duncan's multiple comparisons were used for analyzing differences between groups. The linear and quadratic comparisons were applied to identify the dose-effect of mixed organic acid in broiler chickens.

For microbiota profiling, the raw sequences of 16s rRNA were quality-controlled using fastp (https://github.com/OpenGene/fastp, version 0.20.0) software and spliced by FLASH (http://www.cbcb.umd.edu/software/flash, version 1.2.10) software. The sequences were OTUs clustered and chimeras were removed based on 97% similarity using Uparse software (http://drive5.com/uparse/, version 7.1). Species classification annotation of each sequence was performed using Ribosomal Database Project (RDP) classifier (http://sourceforge.net/projects/rdp-classifier/, version 2.2), comparing to the Silva 16S rRNA database (http://www.arb-silva.de) and setting a comparison threshold at an 0.7 confidence level. The software used to analyze α-diversity (the Shannon and Simpson indices represented the diversity of the intestinal microorganisms, while the Sobs, Ace, and Chao indices reflected the species richness) by mothur (http://www.mothur.org/wiki/, version 1.30.1) and β-diversity was evaluated using principal coordinate analysis (PCoA) by QIIME (University of California, San Diego, USA). Also, the results were plotted with the “vegan” and “ggplot2” packages by R software (Version 3.4.4). The significant differences in microbiological communities between groups were evaluated by ANOSIM with the R package “vegan.” The Linear discriminant analysis (LDA) and effect size (LEfSe) analyses were operated via the LEfSe tool (Shanghai Majorbio Bio-pharm Technology, China). A *P*-value of *P* < 0.05 was considered statistically significant and 0.05 < *P* ≤ 0.10 was indicative of a differential trend.

## Results

### Immune Characteristics

Dietary supplemented with LMOA increased (*P* < 0.05) the content of D-lactate (D-LA) in 21 d-broilers (Table 3). The highest concentrations of IgA, D-LA, and Interlukin-10 (IL-10) were recorded for 42 d-broilers supplementation with HMOA. However, in terms of other serum parameters, there were no significant differences between the treatment and the control group.

**Table 3:** Immune function of serum in 21-day and 42-day-old broilers as affected by dietary MOA supplementation.

**Item**	**Ctrl^a^**	**LMOA**	**HMOA**	**SEM**	***P*** **-value**		
					**ANOVA**	**Linear**	**Quadratic**
**21 d**							
IgA [ug/mL]	11.65	11.33	11.50	0.73	0.95	0.88	0.79
IgG [ng/L]	9.45	9.44	8.83	0.74	0.80	0.57	0.74
D-LA [μmol/L]	5.37^b^	8.87^a^	5.22^b^	0.44	<0.01	0.81	<0.01
DAO [U/mL]	3.23	3.12	2.84	0.16	0.24	0.11	0.67
IL-1β [pg/mL]	88.54	79.67	84.21	5.26	0.52	0.57	0.32
IL-6 [ng/L]	29.02	28.61	27.01	1.38	0.57	0.33	0.73
IL-10 [pg/mL]	12.06	12.46	11.13	0.56	0.27	0.27	0.23
TNF-α [ng/L]	66.41	68.11	63.54	3.91	0.71	0.62	0.53
**42 d**							
IgA [ug/mL]	12.77^b^	13.29^a^^,^ ^b^	14.65^a^	0.48	0.05	0.02	0.49
IgG [ng/L]	10.34	10.81	11.87	0.61	0.24	0.11	0.70
D-LA [μmol/L]	6.51^b^	6.75^a, b^	7.63^a^	0.32	0.05	0.03	0.44
DAO [U/mL]	3.92	3.61	4.02	0.22	0.41	0.74	0.20
IL-1β [pg/mL]	96.03	100.65	105.54	4.68	0.39	0.18	0.98
IL-6 [ng/L]	33.91	33.82	36.86	1.30	0.22	0.14	0.35
IL-10 [pg/mL]	12.81^b^	13.81^b^	16.12^a^	0.48	<0.01	<0.01	0.29
TNF-α [ng/L]	79.63	81.91	87.10	3.66	0.37	0.18	0.75

a−b *In each row, means with the same letter represented no significant differences*.

*^a^Ctrl: a basal diet of corn-soybean meal with 0 mg/kg of mixed organic acid; LMOA: a basal diet with 3,000 mg/kg of mixed organic acids; HMOA: a basal diet with 6,000 mg/kg of mixed organic acids. n = 8*.

*IgA, Immunoglobulin A; IgM, Immunoglobulin M; D-LA, D-lactate; DAO, diamine oxidase; IL-1β, interleukin-1β; IL-6, interleukin-6; IL-10, interleukin-10; TNF-α, tumor necrosis factor-α*.

### Viscera Percentage

Neither on day 21 nor day 42 of broilers were there any differences in spleen, pancreas, or liver weight percentage between the treatment and control (Table 4).

**Table 4:** Offal organ weight in 21 and 42 d of broilers as affected by dietary MOA supplementation (% of body weight).

**Item**	**Ctrl^a^**	**LMOA**	**HMOA**	**SEM**	***P*** **-value**		
					**ANOVA**	**Linear**	**Quadratic**
**21 d**							
Spleen	0.10	0.10	0.08	0.01	0.31	0.21	0.38
Pancreas	0.32	0.30	0.29	0.02	0.58	0.32	0.80
Liver	2.41	2.52	2.43	0.12	0.79	0.89	0.51
**42 d**							
Spleen	0.12	0.14	0.12	0.02	0.75	0.95	0.46
Pancreas	0.17	0.20	0.20	0.02	0.30	0.19	0.40
Liver	1.97	2.09	2.01	0.07	0.48	0.71	0.26

*^a−b^In each row means with the same letter represented no significant differences*.

a *Ctrl: a basal diet of corn-soybean meal with 0 mg/kg of mixed organic acid; LMOA: a basal diet with 3,000 mg/kg of mixed organic acids; HMOA: a basal diet with 6,000 mg/kg of mixed organic acids. n = 8*.

### Intestinal pH Value

The diets supplemented with LMOA and HMOA decreased (*P* < 0.05) the pH values of the duodenum compared to the control and decline linearly (Table 5). However, there were no significant differences in the pH values of the gizzard, jejunum, and ileum.

**Table 5:** The pH value of gut in 42 d-broilers as affected by dietary MOA supplementation.

**Item**	**Ctrl^a^**	**LMOA**	**HMOA**	**SEM**	***P*** **-value**		
					**ANOVA**	**Linear**	**Quadratic**
Gizzard	4.17	3.96	3.86	0.18	0.49	0.25	0.81
Duodenum	5.99^a^	5.64^b^	5.49^b^	0.08	<0.01	<0.01	0.32
Jejunum	5.68	5.96	5.83	0.07	0.06	0.16	0.05
Ileum	6.07	6.23	6.00	0.14	0.31	0.73	0.15

a−b *In each row means with the same letter represented no significant differences*.

*^a^Ctrl: a basal diet of corn-soybean meal with 0 mg/kg of mixed organic acid; LMOA: a basal diet with 3,000 mg/kg of mixed organic acids; HMOA: a basal diet with 6,000 mg/kg of mixed organic acids. n = 8*.

### Antioxidant Enzyme Characteristics of Serum and Small Intestine

**Figure 1:**
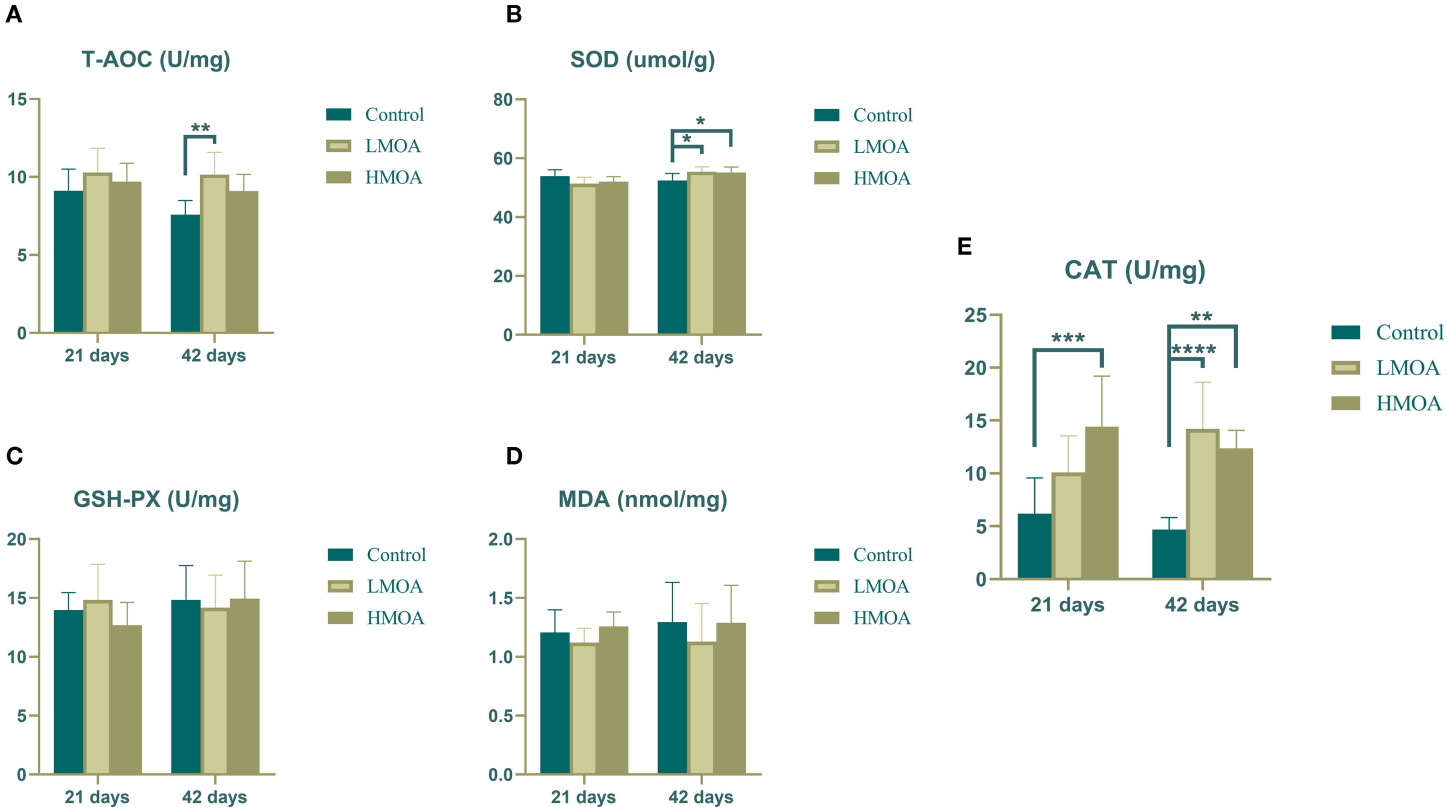
The antioxidant enzyme characteristics of serum in 21 and 42 d-broilers as affected by dietary MOA supplementation. **(A)** Total antioxidant capacity (T-AOC), **(B)** Superoxide dismutase activity (SOD), **(C)** Glutathione peroxidase activity (GSH-Px), **(D)** Malondialdehyde (MDA), **(E)** Catalase activity (CAT). Control: a basal diet of corn-soybean meal with 0 mg/kg of mixed organic acid; LMOA: a basal diet with 3,000 mg/kg of mixed organic acids; HMOA: a basal diet with 6,000 mg/kg of mixed organic acids. Data are indicated as means ± SEM (*n* = 8). Bar with the asterisk (*) level presented the degree of significant difference. ^*^0.01 < *p* < 0.05; ^**^0.001 < *p* < 0.01; ^***^0.0001 < *p* < 0.001; ^****^p < 0.0001.

High (*P* < 0.05) total antioxidant capacity compared to the control was observed (Figure 1) in serum gathered from 42 d-broilers supplemented with the LMOA. The catalase activity of serum in 21 d-broilers fed diets with HMOA was higher than in control. The superoxide dismutase activity and catalase activity were increased (*P* < 0.05) compared to the control at 42 d-broilers fed with LMOA and HMOA, respectively. The total antioxidant capacity and the amount of malondialdehyde were not affected by supplementing with LMOA and HMOA.

**Figure 2:**
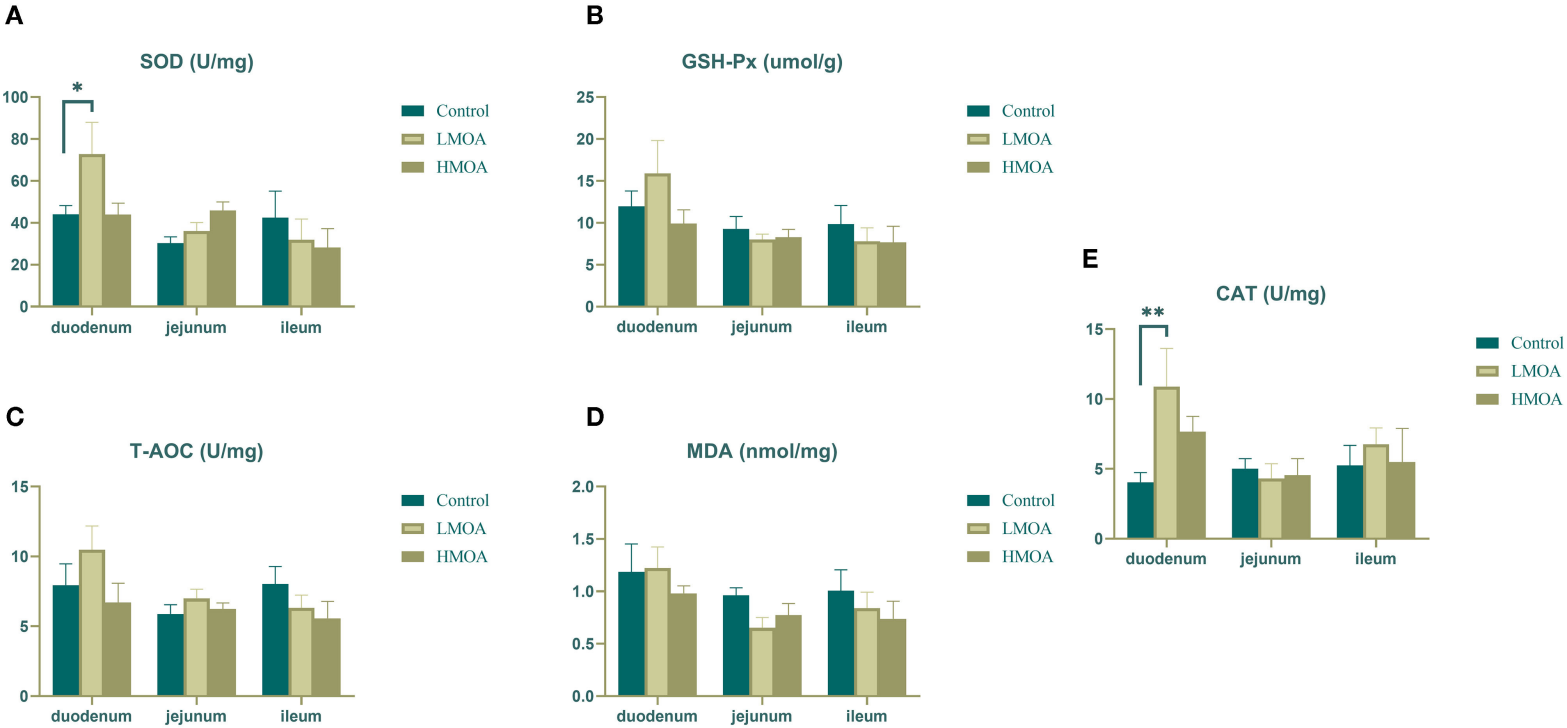
The antioxidant enzyme characteristics of small intestine in 42 d-broilers as affected by dietary MOA supplementation. **(A)** Superoxide dismutase activity (SOD), **(B)** Glutathione peroxidase activity (GSH-Px), **(C)** Total antioxidant capacity (T-AOC), **(D)** Malondialdehyde (MDA), **(E)** Catalase activity (CAT). Control: a basal diet of corn-soybean meal with 0 mg/kg of mixed organic acid; LMOA: a basal diet with 3,000 mg/kg of mixed organic acids; HMOA: a basal diet with 6,000 mg/kg of mixed organic acids. Data are indicated as means ± SEM (*n* = 8). Bar with the asterisk (*) level presented the degree of significant difference. ^*^0.01 < *p* < 0.05; ^**^0.001 < *p* < 0.01.

High (*P* < 0.05) superoxide dismutase activity and catalase activity were found in the duodenum of broilers dietary supplemented with LMOA compared to the control group (Figure 2). The glutathione peroxidase activity, total antioxidant capacity, and amount of malondialdehyde were not influenced in the duodenum, jejunum, and ileum by feeding LMOA and HMOA.

### Digestive Enzyme Activity

**Figure 3:**
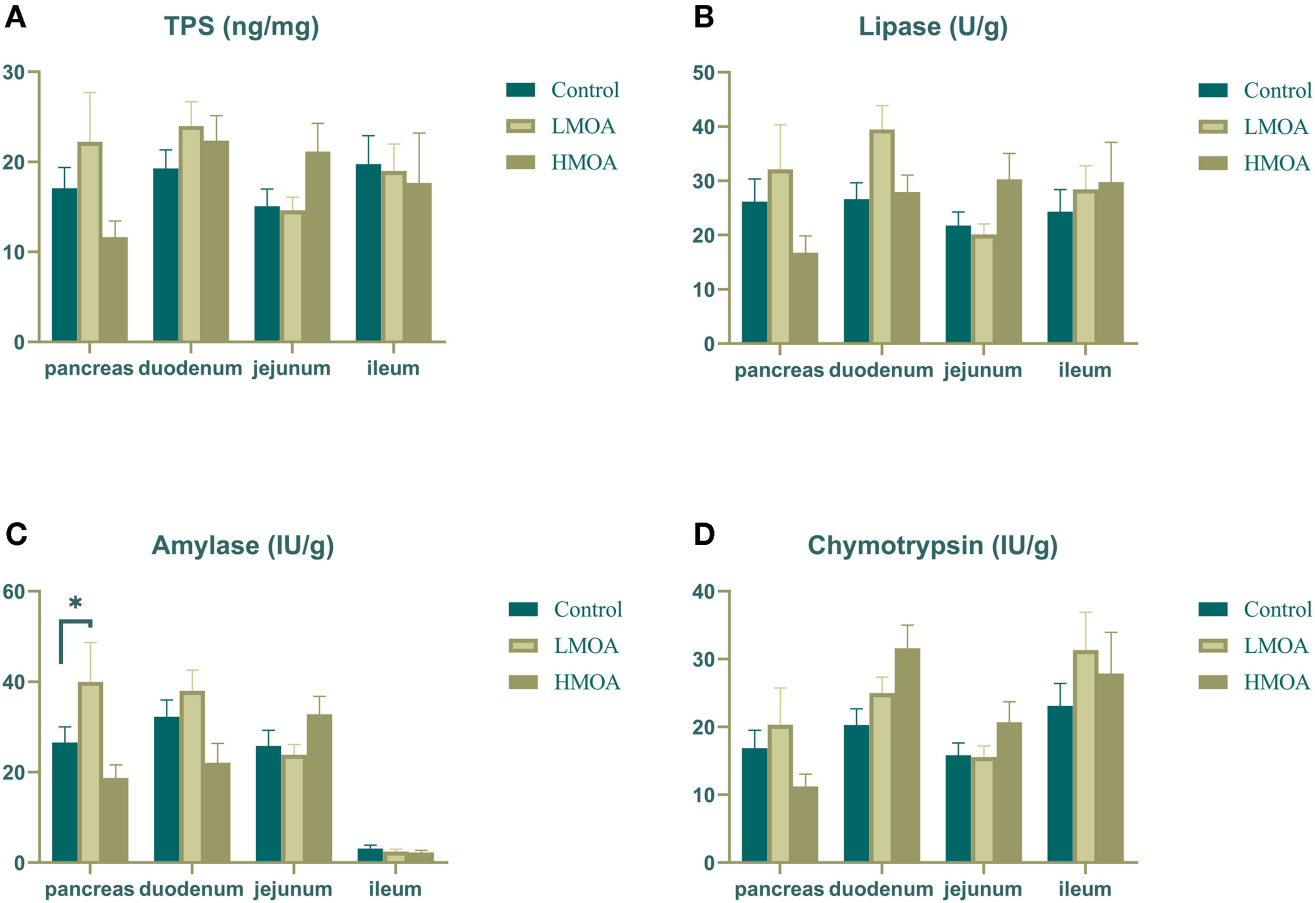
The digestive enzyme activity of pancreas, duodenum, jejunum and ileum in 42 d-broilers as affected by dietary MOA supplementation. **(A)** Trypsin activity, **(B)** Lipase activity, **(C)** Amylase activity, and **(D)** Chymotrypsin activity. Control: a basal diet of corn-soybean meal with 0 mg/kg of mixed organic acid; LMOA: a basal diet with 3,000 mg/kg of mixed organic acids; HMOA: a basal diet with 6,000 mg/kg of mixed organic acids. Data are indicated as means ± SEM (*n* = 8). Bar with the asterisk (*) level presented the degree of significant difference. ^*^0.01 < *p* < 0.05.

The LMOA-supplemented diet increased (*P* < 0.05) the amylase activity in the pancreas of 42 d-broilers (Figure 3). However, the activity of trypsin, lipase, and chymotrypsin in the pancreas, duodenum, jejunum, and ileum of broilers was not observed any significant difference by feeding LMOA and HMOA compared to the control.

### Gene Expression of Tight Junction Protein

**Figure 4:**
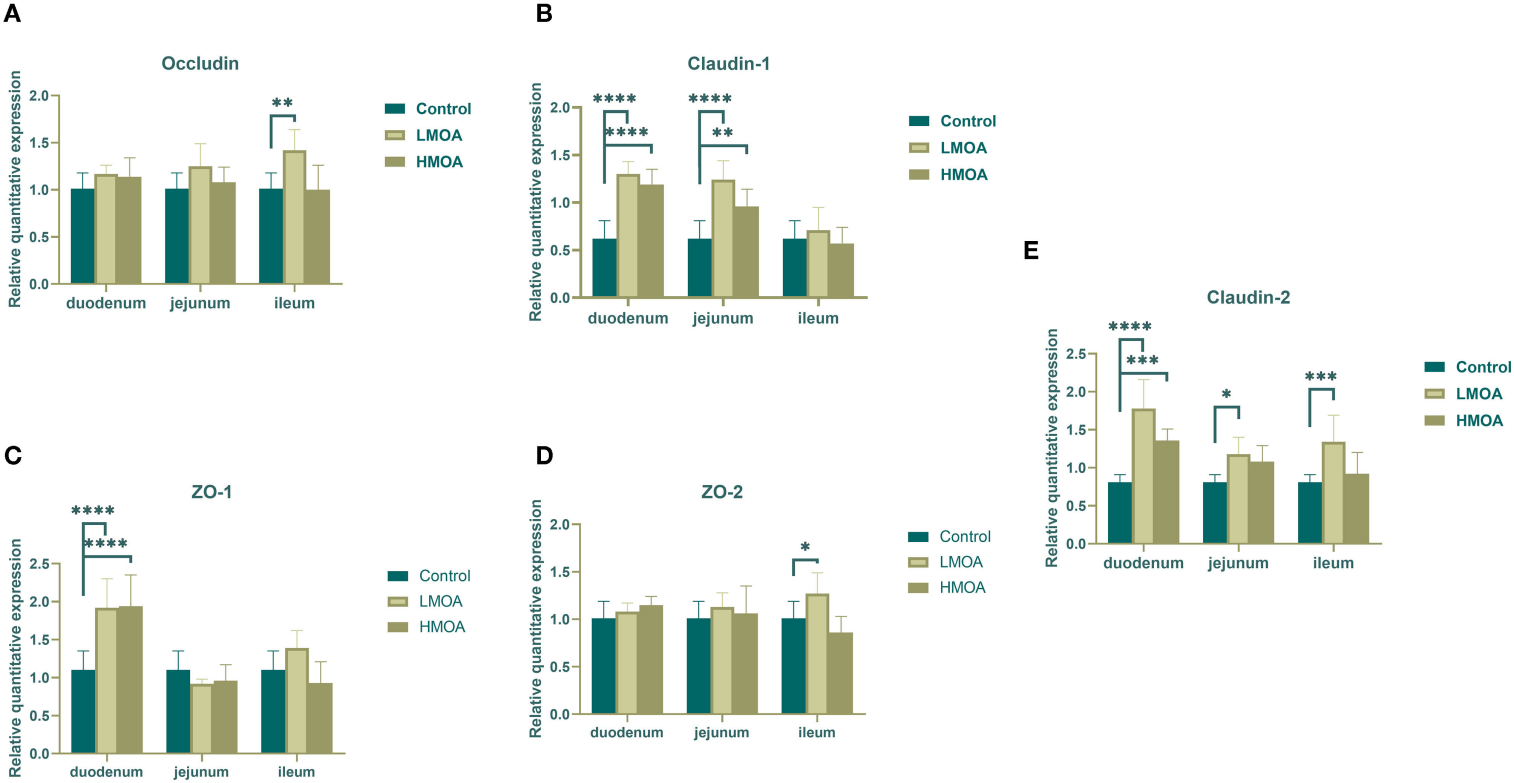
The gene expression of tight junction protein in duodenum, jejunum and ileum of 42 d-broilers as affected by dietary MOA supplementation. **(A)** Occludin, **(B)** Claudin-1, **(C)** ZO-1, and **(D)** ZO-2, **(E)** Claudin-2. Control: a basal diet of corn-soybean meal with 0 mg/kg of mixed organic acid; LMOA: a basal diet with 3,000 mg/kg of mixed organic acids; HMOA: a basal diet with 6,000 mg/kg of mixed organic acids. Data are indicated as means ± SEM (*n* = 8). Bar with the asterisk (*) level presented the degree of significant difference. ^*^0.01 < *p* < 0.05; ^**^0.001 < *p* < 0.01; ^***^0.0001 < *p* < 0.001; ^****^p < 0.0001.

Dietary supplemented with LMOA and HMOA increased (*P* < 0.05) the relative expression of Claudin-1, ZO-1, and Claudin-2 in the duodenum of 42 d-broilers compare to the control group. In addition, LMOA- and HMOA-supplemented diets enhanced (*P* < 0.05) the relative expression of Claudin-1 in the jejunum of 42 d-broilers (Figure 4). The addition of LMOA to the diets caused a higher (*P* < 0.05) gene expression of Claudin-2 in the jejunum and of Occludin, ZO-2, and Claudin-2 in the ileum of 42 d-broilers.

### Intestinal Microbiota Composition

**Figure 5:**
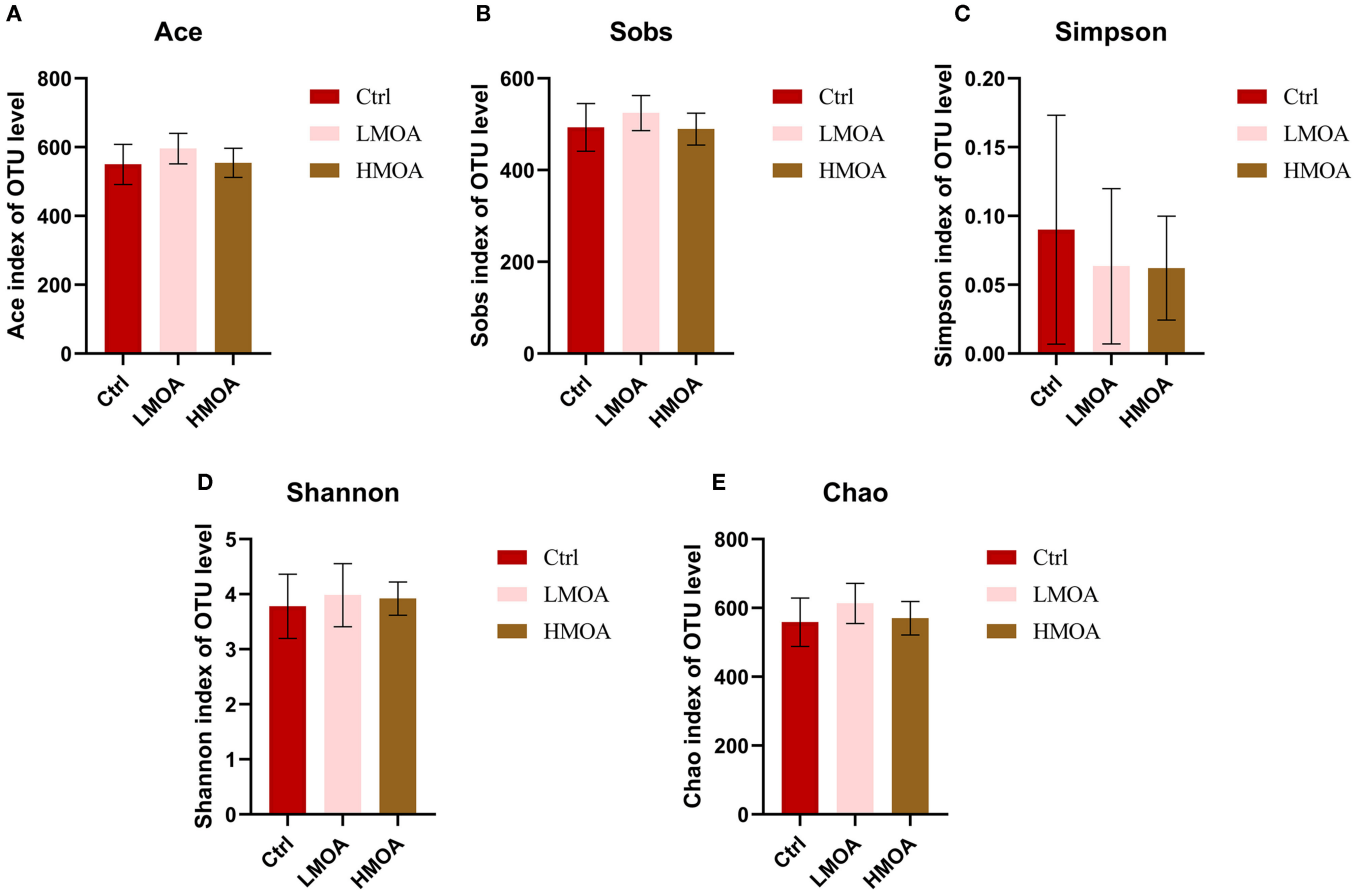
The Bacterial abundance and α-diversity of the cecum in 42 d-broilers dietary with MOA supplementation were assessed by **(A)** Ace index, **(B)** Sobs index, **(C)** Simpson index, **(D)** Shannon index, and **(E)** Chao index of the operational taxonomic units (OTUs) community, respectively. The Shannon and Simpson indices represented the diversity of the intestinal microorganisms, while the Sobs, Ace, and Chao indices reflected the species richness. Ctrl: a basal diet of corn-soybean meal with 0 mg/kg of a mixed organic acid; LMOA: a basal diet with 3,000 mg/kg of mixed organic acids; HMOA: a basal diet with 6,000 mg/kg of mixed organic acids. The values were presented as mean ± standard error by the Kruskal-Wallis H test. *n* = 6.

**Figure 6:**
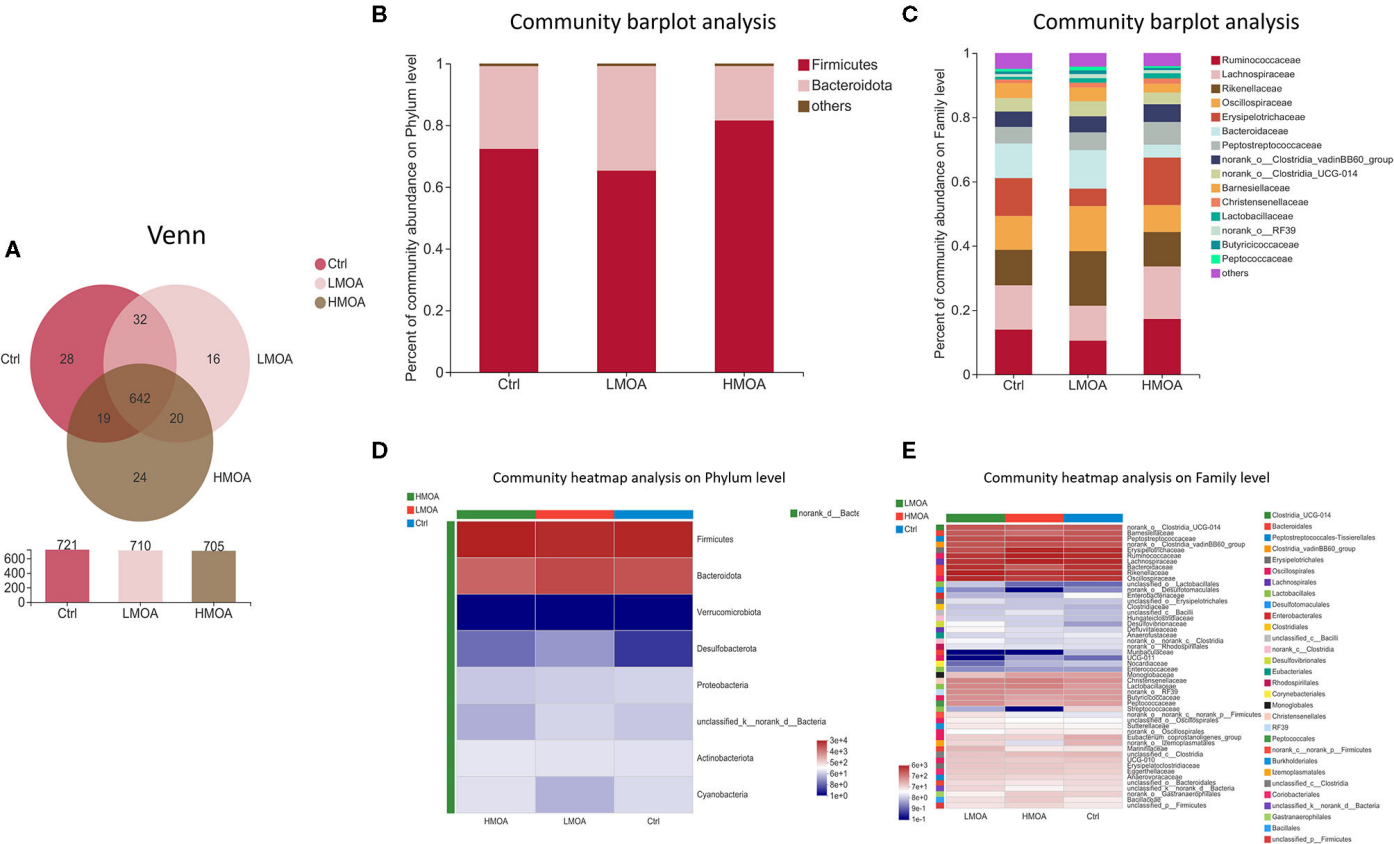
The microbial community composition of cecal in 42-d broilers as affected by dietary MOA supplementation. **(A)** Venn diagrams of dietary treatments at the OTUs level. **(B,C)** Distribution of community barplot in cecum microorganism at the phylum and family level. **(D,E)** Heatmap of the cecum microorganism relative richness at the phylum and family level among dietary treatments. Ctrl: a basal diet of corn-soybean meal with 0 mg/kg of mixed organic acid; LMOA: a basal diet with 3,000 mg/kg of mixed organic acids; HMOA: a basal diet with 6,000 mg/kg of mixed organic acids. *n* = 6.

The bacterial community classification of OTUs was performed on the basis of available sequences with 97% similarity (13). The difference in the α-diversity analysis at OTUs levels between the treatment and the control was not noticed (Figure 5). From the Venn analysis of OTUs in cecum digestive of 42 d-broilers (Figure 6A), there were 28, 16, and 24 unique OTUs in the cecum digestive tract in the control, LMOA, and HMOA groups, respectively, and a total of 642 OTUs were common to all group. At the phylum level (Figures 6B,D), the microbiota composition of cecal contents in 42 d-broilers was primarily *Firmicutes* and *Bacteroidetes*, which accounted for more than 98.5%. The populations of *Firmicutes* in 42 d-broilers fed with HMOA (81.55%) and the populations of *Bacteroidetes* (33.88%) in 42 d-broilers supplemented with LMOA were higher than the control (*Firmicutes:* 72.35%*; Bacteroidetes:* 26.81%)., respectively. At the family level (Figures 6C,E), the microbiota in the cecum digestive of 42 d-broilers were mainly composed of *Ruminococcaceae, Lachnospiraceae, Rikenellaceae, Oscillospiraceae, Erysipelotrichaceae*, and *Bacteroidaceae*, which accounted for beyond 70%.

**Figure 7:**
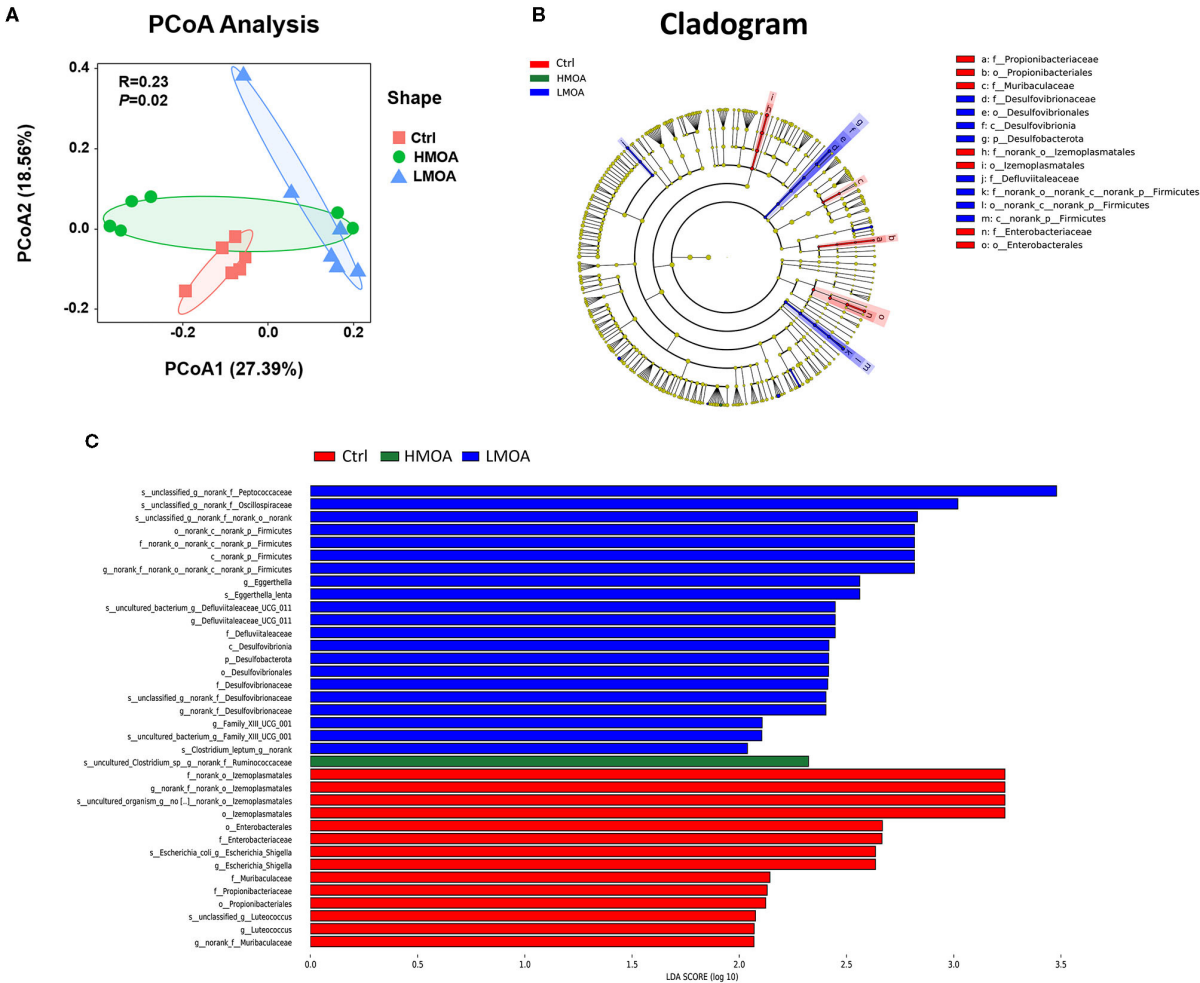
The analysis of bacterial β-diversity and microbial structures in 42 d-broilers cecum as affected by dietary MOA supplementation. **(A)** Principal co-ordinates analysis (PCoA) diagram of the cecum bacterial communities based on bray-Curtis distance calculated from OTUs abundance matrix. The significant difference test between groups using Analysis of similarities (ANOSIM). **(B)** Discriminant analysis of LEfSe multi-level species difference from phylum to genus level. **(C)** Histogram of linear discriminant analysis (LDA) from phylum to genus level. The values were examined by the non-parametric factorial Kruskal-Wallis sum-rank test to identify the microorganisms with significant difference characteristics and using linear discriminant analysis to estimate the magnitude of the effect of abundance on the difference effect for each species. Ctrl: a basal diet of corn-soybean meal with 0 mg/kg of mixed organic acid; LMOA: a basal diet with 3,000 mg/kg of mixed organic acids; HMOA: a basal diet with 6,000 mg/kg of mixed organic acids. *n* = 6.

Principal coordinates analysis (PCoA) based on the distance algorithm of Bray-Curtis calculated from OTUs abundance matrix and the results were examined by statistics acquired from ANOSIM analysis (*R* = 0.23, *P* = 0.02) (Figure 7). The non-parametric factorial Kruskal-Wallis sum-rank test was used to identify the microorganisms with significant difference characteristics and using linear discriminant analysis (LDA threshold > 2.0) to estimate the magnitude of the effect of abundance on the difference effect for each species. The results revealed that the microbiota composition in the cecum was affected by feeding modification as well as the enrichment (*P* < 0.05) of *Desulfovibrionales* order and *Desulfovibrionaceae* family was observed in the cecum of 42 d-broilers diets with LMOA supplementation. Furthermore, the higher relative abundance of *Enterobacteriaceae* order and *Enterobacterales* family were noticed (*P* < 0.05) in the control group.

## Discussion

The previous study has demonstrated that dietary supplemented with mixed organic acid could improve performance (14), immune function (5, 15), intestinal morphology as well as intestinal microbiota (10, 16). Also, numerous scholars have illustrated the mechanism of MOA and summarized the benefits of MOA in livestock and poultry (4, 17), especially in weaning pigs (7, 8). The inconsistent results often occurred concerning the effects of MOA, although MOA always performed a positive impact (18, 19). In our paper, we focused on the serum immune function, antioxidant capacity, pH value, tight junction, and microbiota of intestine in 21 and 42 d broilers in terms of their being affected by dietary supplemented with MOA at different levels, which would be helpful in determining the appropriate level of MOA and in confirming whether MOA plays a role on intestine regarding antioxidant systems, digestive enzyme activity, mechanical barrier, etc.

Lactate is a metabolite of the anaerobic oxidation of glucose (glycolysis) and an essential oxidant of the aerobic metabolic system. It is primarily generated in bone, muscle, brain, and red blood cells. It is transformed into glucose in the liver via the gluconeogenic pathway, which plays a vital role in the energy supply system (20). Measurement of serum lactate level indicates the state of tissue oxygen supply and metabolism as well as inadequate perfusion (21, 22). However, excessive levels of lactic acid have a negative effect on the acid-base balance of the organism and trigger metabolic diseases (23). A higher level of D-lactate in the serum of 21 and 42 d-broilers dietary with HMOA supplementation was noticed in our present study, which presumably associated with the composition of the mixed organic acid product containing lactic acid and the concentration of mixed organic acid.

IgG, IgA, and IgM are three essential immunoglobulins, and the immune status could be determined by measuring the level of immunoglobulins in the serum (24). IL-10 is a cytokine with pleiotropic properties in immune regulation and inflammation. It downregulates the expression of Th1 cytokines, MHC-II class antigens, and co-stimulatory molecules on macrophages (25). Likewise, it enhances the survival, proliferation, and antibody production of B cells. IL-10 blocks NF-κB activity and is involved in the regulation of the JAK-STAT signaling pathway (26). Our findings that dietary HMOA supplementation increased the IgA level were similar to the previous research that mixed organic acid and medium-chain fatty acids combined linear increased the concentration of IgG in serum of broiler. The differences may be connected to the ingredients of the diet, the composition, and the combination of the organic acid mix and the broiler breed. It is poorly understood concerning the increase in serum concentrations of IL-10. Still, the latest findings demonstrated that SCFAs increased IL-10 concentrations in T cells and regulatory B cells by inhibiting histone deacetylase from enhancing histone acetylation and regulating the mammalian target of rapamycin (mTOR) (27, 28).

The development of the spleen, as an immune organ of poultry, was used as an indicator to evaluate immune performance (29). The pancreas performs a critical role in the process of digestion and absorption of feed nutrients in poultry. The development of the liver is an indicator to assess the metabolic function of poultry. In the present research, the difference was not observed between control and treatments in the spleen, pancreas, and liver of 21 and 42 d-broilers, which inconsistent with the Mohamed et al. (30), they indicated that the broilers fed with organic acid increased the weight of the spleen, the findings of Abdel-Fattah et al. (31) and Ghazalah et al. (32) also supported the conclusion. The reason for the discrepancy is probably related to the composition of the mixed organic acids as well as their concentrations, the specific reason for which is obscure, which needed to be confirmed further.

The digestive tract of poultry is comparatively short, especially in juvenile animals, and the pH of the small intestine is vulnerable to external environmental factors (4). In principle, mixed organic acids are capable of assisting the digestive tract in maintaining a suitable acidity, which also acts as one of the fundamental mechanisms of mixed organic acids (3, 33). Nevertheless, the results that mixed organic acids generally reduce the pH value of the gastrointestinal tract of animals were widely varied. Ndelekwute et al. (34) demonstrated that the addition of 0.25% acetic acid and butyric acid to broiler diets, respectively decreased the pH value of the duodenum and cecum of broilers. However, it has also been found that the diets supplemented with acetic acid, butyric acid, citric acid, or lactic acid had no significant effect on the pH value of the duodenum, jejunum, and ileum of broilers (35). In our findings, diets with LMOA and HMOA supplementation decreased the pH value of the duodenum in broilers, but unaffected on other digestive tracts, which indicated that the effect of mixed organic acids on small intestinal pH was mainly centered in the anterior segment. Still, the results were inconsistent with Paul et al. (36) and Giannenas et al. (37), they reported that pH remained unaffected supplemented with organic acid salt and benzoic acid combined with essential oil, respectively. The differences may be associated with the metabolism of the mixed organic acid with the consumption of chyme in the digestive tract (35) as well as a possibility that the commercial feeds were formulated with a high buffering capacity to be compatible with the nutritional and energy requirements of modern broilers (38).

The pancreas of poultry exerts trypsin, chymotrypsin, pancreatic amylase, and pancreatic lipase, with amylase, protease, and lipase being the majority and only functioning after entering the small intestine (39). Hence, improved digestibility can be predicted to occur with higher digestive enzyme activity in the intestine or the gut's capability to absorb nutrients (40). In the present study, dietary supplementation with LMOA increased the amylase activity of the pancreas in broilers but at the level of intestinal digestion. No LMOA or HMOA had an impact on the trypsin, lipase, and chymotrypsin activities, which is in line with a study by Palamidi et al. (38), who reported that diets with mixed organic acid did not show an observed difference in the amylase and lipase activities of the small intestine and even decreased the trypsin activity of pancreas in broilers. The results were perhaps associated with the feedback inhibition of proteases in the duodenum according to Li et al. (41). Similarly, the addition of lactic acid to diets did not show any change in digestive enzymes in the pancreas and small intestine of broilers (42). The increased activity of pancreatic lipase may be correlated with the lower pH of the gastrointestinal tract (43), which was required to confirm further.

Oxidative stress, one of the primary pathological factors affecting animal growth performance (44), is a state of imbalance in the oxidative system owing to the excessive generation and accumulation of reactive oxygen species (ROS) (45). The level of malondialdehyde, as a metabolite of the peroxidation reaction between free radicals and biofilm lipids, directly reflects the degree of tissue peroxidation and indirectly reflects the degree of cell damage caused by oxygen-free radicals. Consequently, animals are well-equipped with basic anti-ROS defense systems to maintain ROS homeostasis (46). Superoxide dismutase (SOD) is an essential antioxidant enzyme that catalyzes the disproportionation of ${\text{O}}_{2}^{-}$ to O_2_ and H_2_O_2_ and the removal of ${\text{O}}_{2}^{-}$ from the mitochondrial membrane and mitochondrial matrix, often regarded as an indicator of the ability to respond to oxidative stress. Glutathione peroxidase (GSH-Px) is involved in converting between reduced glutathione and oxidized glutathiol, reduced H_2_O_2_ to H_2_O, and lipid peroxides to alcohols. Total antioxidant capacity (T-AOC) was used as an indicator of the overall antioxidant capacity of the organism (47, 48). In our research, diets with HMOA supplementation enhanced the content of SOD and CAT in the serum of 21 and 42 d-broiler, respectively, and the level of T-AOC, SOD, and CAT were increased in serum of 42 d-broiler fed with LMOA. Additionally, an improvement of the SOD and CAT were observed in the duodenum of broilers supplemented with LMOA, which corresponded with Abudabos et al. (49), who reported that dietary organic acid enhanced the T-AOC and lowered the concentration of H_2_O_2_ in serum to reduce the oxidative stress status of 42 d-broilers. Likewise, diets supplemented with organic acid and *Bacillus subtilis* combination improved the concentration of T-AOC in the serum of broilers challenged with *Salmonella typhimurium* (50). Besides, citric acid and malic acid were discovered to have a robust antioxidative capacity by Tezcan et al. (51). However, with the exception of the duodenum, there was no significant modification of the antioxidant index in the jejunum and ileum, which was probably attributed to the fact that the mixed organic acids only served in the stomach and duodenum and were unable to completely reached and act in the posterior intestinal tract (3, 33). Therefore, it is desirable to upgrade the production technology (e.g., microencapsulation), making it available for the mixed organic acids to perform continuously and stably in the entire intestinal segment of broilers as well as improving the antioxidant function of broilers.

The function of the intestinal mechanical barrier is a precondition for upholding the homeostasis of mucosal function while sustaining the capacity to absorb nutrients (52). The tight junction is the primary intercellular junction positioned at the tip of the epithelium and surrounding the cells in a hoop-like fashion, which tightly connects adjacent epithelial cells, preventing the passage of toxic macromolecules and microorganisms and defending the intestinal mucosal barrier. The tight junction was classified into structural and functional proteins, the principal structural proteins are occludin, Claudin and junction adhesion molecule (JAM) and the main functional proteins are Z0-1, Z0-2, Z0-3, Cingulin, and Zonulin (53). In this work, we demonstrated that dietary supplementation with the LMOA and HMOA enhanced the expression of tight junction (mainly Claudin-1, Claudin-2, and ZO-1) in the small intestine, respectively; to some extent, the broilers fed with LMOA performed better than the HMOA group. Identical conclusions were obtained by Yang et al. (54), they indicated that dietary supplemented encapsulated organic acids increased the Claudin-1 mRNA expression, and numerous studies have also been verified in other animals that the expression of tight junction proteins in the small intestine of animals was enhanced by organic acids (55–58). Therefore, mixed organic acids regulated the mechanical barrier of the animal intestine and defended the intestine from exposure to direct pathogenic bacteria mainly by elevating the expression of genes associated with tight junction proteins Claudin, Occludin, and Z0s in the intestine.

The intestinal microorganisms performed a key nutritional, immune, and regulatory role in host metabolism, and dysbiosis of the bacteria triggered a range of diseases. For example, acute enteritis has been proved to be correlated with specific pathogenic intestinal dominant bacteria (59). Variations in the intestinal microorganisms were indicative of the status of the intestinal bacteria. The results of our present study revealed that the supplementation of diets with mixed organic acids seems to be dose-dependent on the intestinal microbial structure of broiler cecum, which should be validated with statistics analysis.

Normally, the diversity of microorganisms is positively correlated with the capacity to equilibrate the structure of the intestinal microorganisms and to resist colonization by foreign pathogens. The diversity and richness of the intestinal microorganisms decrease when the organism undergoes disease (60). In our paper, diet supplementation with LMOA increased the Chao, Shannon, Sobs, and Ace indices, but this did not show a significantly observable difference; the differences occurred may be associated with the breeds and ages of broilers as well as the composition of mixed organic acids (61).

It has been shown that *Bacteroidetes* and *Firmicutes* constitute the majority of the microbial community at the phylum level in broilers, which performed a paramount role in energy production and metabolism (62–64). Notably, *Firmicutes* has been reported as the dominant phylum in the chicken cecum community (65, 66), while *Bacteroidetes* was also reported as the dominant phylum (64, 67). In this study, there was no significant difference in the relative abundance of broiler cecum microorganisms at the phylum level among the treatments. The *Bacteroidetes* and *Firmicutes* were the dominant bacteria, accounting for more than 98.5% of the total microbial community, and the *Firmicutes* occupied the maximum percentage. The reasons for the differences that appeared might be attributed to the age and breed of the selected chickens as well as regional differences (68), which also indicated that the microbiota of the broiler cecum was being modified.

*Ruminococcaceae* are capable of generating short-chain fatty acids, which could inhibit the growth and reproduction of pathogenic intestinal bacteria by impacting the intestinal pH, which is a prerequisite and guarantee for achieving a good growth performance in broilers (69, 70). This is in accordance with our findings of the enhanced relative abundance of *Ruminococcaceae* in cecum broilers fed with HMOA. Sulfate-reducing bacteria of the family *Desulfovibacteriaceae* consume short-chain fatty acids, which are crucial for intestinal epithelial cells, caused damage to intestinal epithelial cells by producing H_2_S (71–73), and their lipopolysaccharides have a stronger ability to trigger inflammation (74). So, *Desulfovibacteriaceae* might be a functional bacterium with a vital role in the intestine and possibly played an essential character in developing metabolic syndrome. Consequently, the increased relative abundance of *Desulfovibacteriaceae* in the LMOA group in this study may not be a favorable phenomenon, an occurrence that requires further confirmation. In addition, a higher relative abundance of *Escherichia coli* was noted in the control group, which indicated diet supplemented with mixed organic acid promoted intestine health by reducing harmful pathogenic bacteria, which were identified by numerous broilers studies (19).

## Conclusions

In summary, our findings demonstrated the beneficial impacts of the broiler dietary supplementation with mixed organic acid enhanced the immune characteries and antioxidative function in serum and small intestine, promoted the digestive enzyme activity of the pancreas, improved the expression of tight junction proteins, and modulated the cecum bacterial community, which achieves a better healthy growth for broilers. However, there are still some unknown questions remaining to be solved such as the increased relative abundance of *Desulfovibacteriaceae* in cecum broilers diets with LMOA. Therefore, additional relevant research may contribute to the resolution of this phenomenon. In addition, considering the overall effect of the experiment and the economic cost, it is feasible to supplement the diet with mixed organic acid at 3,000 mg/kg to promote the antioxidant characteristics and health status of broilers.

## Data Availability Statement

The original contributions presented in this study are included in the article/supplementary material, further inquiries can be directed to the corresponding author/s.

## Ethics Statement

The animal study was reviewed and approved by the Institutional Animal Care and Use Committee of China Agricultural University (No.AW09089104-1, Beijing, China).

## Author Contributions

JM, XP, and SM: conceptualization. JM, XP, and JW: methodology. JM: software, investigation, data curation, and writing—original draft preparation. JM, SM, and JW: validation and resources. JM and JW: formal analysis. SM and XP: writing—review and editing, and supervision. XP: project administration and funding acquisition. All authors contributed to the article and approved the submitted version.

## Conflict of Interest

The authors declare that the research was conducted in the absence of any commercial or financial relationships that could be construed as a potential conflict of interest.

## Publisher's Note

All claims expressed in this article are solely those of the authors and do not necessarily represent those of their affiliated organizations, or those of the publisher, the editors and the reviewers. Any product that may be evaluated in this article, or claim that may be made by its manufacturer, is not guaranteed or endorsed by the publisher.
